# Measurement of immunofunctional leptin to detect and monitor patients with functional leptin deficiency

**DOI:** 10.1530/EJE-16-0821

**Published:** 2016-12-22

**Authors:** Martin Wabitsch, Lutz Pridzun, Michael Ranke, Julia von Schnurbein, Anja Moss, Stephanie Brandt, Katja Kohlsdorf, Barbara Moepps, Michael Schaab, Jan-Bernd Funcke, Peter Gierschik, Pamela Fischer-Posovszky, Bertram Flehmig, Jürgen Kratzsch

**Affiliations:** 1 Division of Pediatric Endocrinology and DiabetesDepartment of Pediatrics and Adolescent Medicine, University Medical Center Ulm, Ulm, Germany; 2 Mediagnost GmbHReutlingen, Germany; 3 Division of Pediatric Endocrinology and DiabetesDepartment of Pediatrics and Adolescent Medicine, University of Tübingen, Tübingen, Germany; 4 Institute of Pharmacology and ToxicologyUniversity Medical Center Ulm, Ulm, Germany; 5 Institute of Laboratory MedicineClinical Chemistry and Molecular Diagnostics, University of Leipzig, Leipzig, Germany

## Abstract

**Context and aims:**

Functional leptin deficiency is characterized by high levels of circulating immunoreactive leptin (irLep), but a reduced bioactivity of the hormone due to defective receptor binding. As a result of the fact that affected patients can be successfully treated with metreleptin, it was aimed to develop and validate a diagnostic tool to detect functional leptin deficiency.

**Methods:**

An immunoassay capable of recognizing the functionally relevant receptor-binding complex with leptin was developed (bioLep). The analytical quality of bioLep was validated and compared to a conventional assay for immune-reactive leptin (irLep). Its clinical relevance was evaluated in a cohort of lean and obese children and adults as well as in children diagnosed with functional leptin deficiency and their parents.

**Results:**

In the clinical cohort, a bioLep/irLep ratio of 1.07 (range: 0.80–1.41) was observed. Serum of patients with non-functional leptin due to homozygous amino acid exchanges (D100Y or N103K) revealed high irLep but non-detectable bioLep levels. Upon treatment of these patients with metreleptin, irLep levels decreased, whereas levels of bioLep increased continuously. In patient relatives with heterozygous amino acid exchanges, a bioLep/irLep ratio of 0.52 (range: 0.48–0.55) being distinct from normal was observed.

**Conclusions:**

The new bioLep assay is able to diagnose impaired leptin bioactivity in severely obese patients with a homozygous gene defect and in heterozygous carriers of such mutations. The assay serves as a diagnostic tool to monitor leptin bioactivity during treatment of these patients.

## Introduction

The leptin/leptin receptor system is crucial for the regulation of body weight (1, 2). Leptin is mainly produced in white adipocytes and secreted into the circulation (1, 3, 4). Leptin reflects the body’s energy stores in adipose tissue and regulates body energy homeostasis as well as adipose tissue mass in a complex circuit involving central nervous pathways. Inborn errors of the leptin/leptin receptor system in humans are rare conditions caused by mutations in either the leptin or the leptin receptor gene that lead to early onset extreme obesity (2, 5, 6, 7, 8). Beginning in infancy, affected children show an insatiable appetite and hyperphagia resulting in an extreme debilitating, often life-threatening obesity and hypogonadotropic hypogonadism at pubertal age (5, 6, 9).

In serum of patients with leptin deficiency, no detectable leptin can be measured by conventional immunoassays. Fortunately these patients can be treated efficiently with recombinant human methionyl-leptin (metreleptin) to normalize their eating behavior, reduce their fat mass, correct metabolic alterations and initiate gonadotropin pulses in pubertal or post-pubertal patients (5, 7, 9).

Recently, the new entity of functional leptin deficiency has been described (10, 11). The clinical presentation of patients with bioinactive leptin is similar to that of patients with classical leptin deficiency. In these patients, circulating leptin levels measured by standard immunoassay appear to be appropriate for their fat mass. However, the mutated leptin does not bind to the leptin receptor due to a structural alteration and is thus bioinactive.

As patients with biologically inactive leptin can be treated with recombinant human leptin similar to patients with classical leptin deficiency (10, 11), we aimed to establish and to validate a diagnostic tool to detect functional leptin deficiency. Here, we present the analytical characteristics of this assay and the results of its clinical validation. We suggest that it may serve as a diagnostic tool as well as a fast and cheap alternative to gene sequencing in the workup of patients suspected of having biologically inactive leptin.

## Subjects and methods

### Cohort for clinical validation of the bioLep assay

For the clinical validation of the assay, we used frozen serum samples from a cohort of normal-weight as well as obese but otherwise healthy children and adults recruited from Southern Germany: *n* = 409; 52% females; age (interquartile range IQR: 9.4–16.4 years); body mass index BMI (IQR: 19.1–41.9 kg/m^2^); BMI standard deviation score BMI-SDS (IQR: 0.77–3.4). Written informed consent was obtained from adults and from parents of enrolled children. The ethics committee of the University of Ulm approved this study. The inclusion of human subjects in this study complies with the Declaration of Helsinki.

### Patients with bioinactive leptin or leptin deficiency

In this study, three patients (patients A, B and C) with biologically inactive leptin as well as two patients with classical leptin deficiency (patients D and E) have been included. Clinical characteristics of the patients with biologically inactive leptin have been published earlier (10, 11). At the time of their first visits, we have seen a boy (patient A) at the age of 2 years and 6 months with a BMI of 44.6 kg/m^2^ who had a D100Y mutation, a boy (patient B) at the age of 6 years with a BMI of 35.2 kg/m^2^ and a girl (patient B) at the age of 9 years with a BMI of 39.6 kg/m^2^ who both had a N103K mutation in the leptin gene.

The two patients with classical leptin deficiency were a boy (patient D) with a R105W mutation and a girl (patient E) who had a L72S mutation. Clinical data of the boy have not been published yet, whereas clinical data of the girl have been already published (12).

The three patients (patient A, B and C) with biologically inactive leptin as well as the two patients with classical leptin deficiency (patient D and E) were treated with recombinant human methionyl-leptin (metreleptin). Data on bioLep and irLep concentrations, percent fat mass (% FM) by DEXA, and metreleptin dosage (mg/kg lean body mass per day) of the treated patients are available for the following patients and time points: patient A at start of treatment (T0) and on treatment after 2, 8 and 23 months (T1–T2–T3), for patient B at the start of treatment (T0), and on treatment after 1, 2 and 3 months (T1–T2–T3), for patient C at the start of treatment (T0), and on treatment after 1, 2 and 10 months (T1–T2–T3); for patient D at start of treatment (T0), and on treatment after 1, 6 and 15 months (T1–T2–T3 and for patient E at the start of treatment (T0) and on treatment after 1, 11 and 22 months (T1–T2–T3).

### Production of leptin-containing HEK293 medium supernatants

The wild-type (WT) leptin coding sequence (translated portion of NCBI reference sequence NM_000230.2) was amplified from human adipocyte cDNA using PCR primers incorporating a 5′ *KpnI* restriction site, blunt-end cloned into pCR-Blunt using the Zero Blunt PCR Cloning Kit (Life Technologies) according to the manufacturer’s instructions and then transferred to pcDNA3.1(+) (Life Technologies) by sticky-end cloning using *KpnI* and *EcoRV*. To generate functionally inactive leptin variants (D100Y or N103K mutated leptin), point mutations were introduced into the wild-type sequence of pcDNA3.1 (+)-leptin WT using the QuikChange II Site-Directed Mutagenesis Kit (Agilent Technologies) according to the manufacturer’s instructions.

For the production of leptin-containing medium supernatants, HEK293 cells were transfected with the respective pcDNA3.1 (+)-leptin WT, -leptin D100Y or -leptin N103K plasmid using jetPRIME (VWR, Darmstadt, Germany) according to the manufacturer’s instructions. Supernatant containing leptin variants was used for testing the functionality of the bioLep assay. Briefly, for each transfection 4 × 10^6^ cells were seeded in a 10 cm dish and left to adhere overnight. Transfections were performed using 8 µg of plasmid DNA and 16 µL of jetPRIME reagent per dish. Media supernatants were harvested 36 h after transfection, cleared from cells by centrifugation and stored at −80°C until measurement.

### Assays

#### Immunoreactive leptin (irLep) measurement

Immunoreactive leptin (irLep) measurement was performed using either the ‘human leptin ELISA’ or the ‘human sensitive leptin ELISA’ (both from Mediagnost, Reutlingen, Germany, www.mediagnost.de) according to the respective kit’s instructions. In brief, the assays are sandwich type ELISAs that employ two monoclonal anti-leptin antibodies as solid phase and detection antibodies, respectively. The intra-assay coefficients of variation (CVs) are ≤5.2% and ≤4.4%, inter-assay CVs are ≤19.2% and ≤7.2%, the least-detectable concentrations are <0.25 ng/mL and <0.01 ng/mL, respectively. The assays are calibrated according to the WHO International Standard for human leptin, NIBSC 97/594.

#### Soluble leptin receptor (sLep-R) measurement

Soluble leptin receptor (sLep-R) measurement was performed using the ‘human sLep-R ELISA’ (Mediagnost) according to the kit’s instructions. This sandwich type ELISA uses two monoclonal anti-sLep-R antibodies as solid phase and as detection antibody, respectively. The intra- and inter-assay CVs are ≤5.9% and ≤10.9%, respectively, the least-detectable-concentration is <0.01 ng/mL. The assay is calibrated against the purified recombinant extracellular domain of the human leptin receptor as described for the bioLep assay.

#### Immunofunctional leptin (bioLep) measurement

The bioLep ELISA (Mediagnost) detects functional leptin in serum according to the principle described by Kratzsch *et al*. (13). In detail, microtiter plates were coated with the purified recombinant extracellular domain of the human leptin receptor. The extracellular domain of the human leptin receptor (UniProt accession No. P48357) was expressed in HEK293 cell cultures as a chimeric molecule fused to a human IgG1-Fc (Mediagnost) and subsequently coupled as a homodimer to the solid phase. Added functional serum leptin is bound to the immobilized receptor and is subsequently detected by a highly specific polyclonal, biotin-conjugated anti-leptin antibody and a streptavidin-peroxidase conjugate. Non-receptor-binding leptin variants give no signal. The assay is calibrated according to the WHO International Standard for human leptin, NIBSC 97/594.

### Statistical analysis

The descriptive statistics report interquartile range (IQR), median, mean and standard deviation for continuous variables (age, BMI, BMI-SDS, bioLep, irLep, sLep-R and bioLep/irLep) and numbers and proportions for categorical variables (sex) in the cohort for clinical validation of the bioLep assay. Non-linear regression analyses (log–log, least squares fit) were conducted to assess the association between sLep-R, irLep and bioLep concentrations in the cohort for clinical validation of the bioLep assay. Statistical analyses were performed with GraphPad Prism, version 6.01 (GraphPad Software).

## Results

### Characteristics of the bioLep assay

#### Quality criteria

The new assay specifically detects and measures leptin molecules capable of binding to the extracellular domain of the leptin receptor. The linear range of the standard dose–response-curve covers 0.05–4 ng/mL (mean of *n* = 10 different runs: *y* = 0.46*x* + 0.006, *r*^2^ = 0.999). Samples with levels below the linear range of the assay were not included. Sample dilution is linear from 1:10 up to 1:160 (4 different samples with levels from 9.9 to 52.9 ng/mL, *r*^2^ > 0.98). Its intra- and inter-assay CVs are ≤4.8% (16-fold determination in each of 6 different serum samples with leptin levels ranging from 2.5 to 54.5 ng/mL) and ≤10.7% (15 determinations in 17 different samples with leptin levels ranging from 2.6 to 41.3 ng/mL), respectively. The mean recovery rates of leptin IS NIBSC 97/594 and metreleptin in serum were 103.2% and 100.1%, respectively (3 different samples with levels from 11.5 to 47.5 ng/mL, each spiked with 20, 40 and 80 ng/mL leptin IS or metreleptin).

#### Proof of principle

Using bioLep and irLep assays, we measured leptin concentrations in media supernatants of HEK293 cells overexpressing either wild-type leptin or the D100Y and N103K mutants, which are incapable of binding to the leptin receptor (10, 11). As shown in Table 1, irLep measurements were not suspicious for both the D100Y and the N103K leptin mutants, whereas the bioLep assay yielded values at the lower limit of detection. Thus, the bioLep assay is able to discriminate between wild-type leptin and mutant leptin with defective receptor binding.

**Table 1:** Concentrations of biologically active leptin (bioLep) and immunoreactive leptin (irLep) in media supernatants of HEK293 cells overexpressing wild-type leptin or the leptin mutants D100Y and N103K.

	bioLep (ng/mL)	irLep (ng/mL)	bioLep (%)
HEK293 supernatants			
WT sample 1	499.6	557.3	89.6
WT sample 2	128.3	143.3	89.5
D100Y	<0.5	160.7	n.a.
N103K	<0.5	32.5	n.a.

n.a.: not available as the bioLep was below the linear assay range.

#### Potential interference of endogenous soluble leptin receptor (sLep-R) with bioLep

The recovery of leptin at concentrations of 10 and 50 ng/mL (IS NIBSC 97/594) as measured by irLep and bioLep after adding increasing concentrations of soluble leptin receptor between 0 and 150 ng/mL was tested. The recovery rates were comparable in both assays (Table 2). Within the physiological range of sLep-R concentrations found in normal weight and obese children (up to 50 ng/mL) (14), no clinically relevant interference was detected.

**Table 2:** Recovery of bioLep and irLep upon adding increasing concentrations of sLep-R to either 10 ng/mL or 50 ng/mL of recombinant leptin IS NIBSC 97/594 (target value).

	Recovery (%)			
	Leptin 10 ng/mL		Leptin 50 ng/mL	
sLep-R (ng/mL)	bioLep	irLep	bioLep	irLep
0	100.0	100.0	100.0	100.0
20	99.4	93.8	100.1	101.5
40	92.1	88.0	94.7	98.4
60	92.7	82.4	96.1	98.4
80	90.5	81.6	93.1	98.4
100	89.0	78.1	93.8	95.2
150	79.7	72.9	91.5	91.3

**Figure 1:**
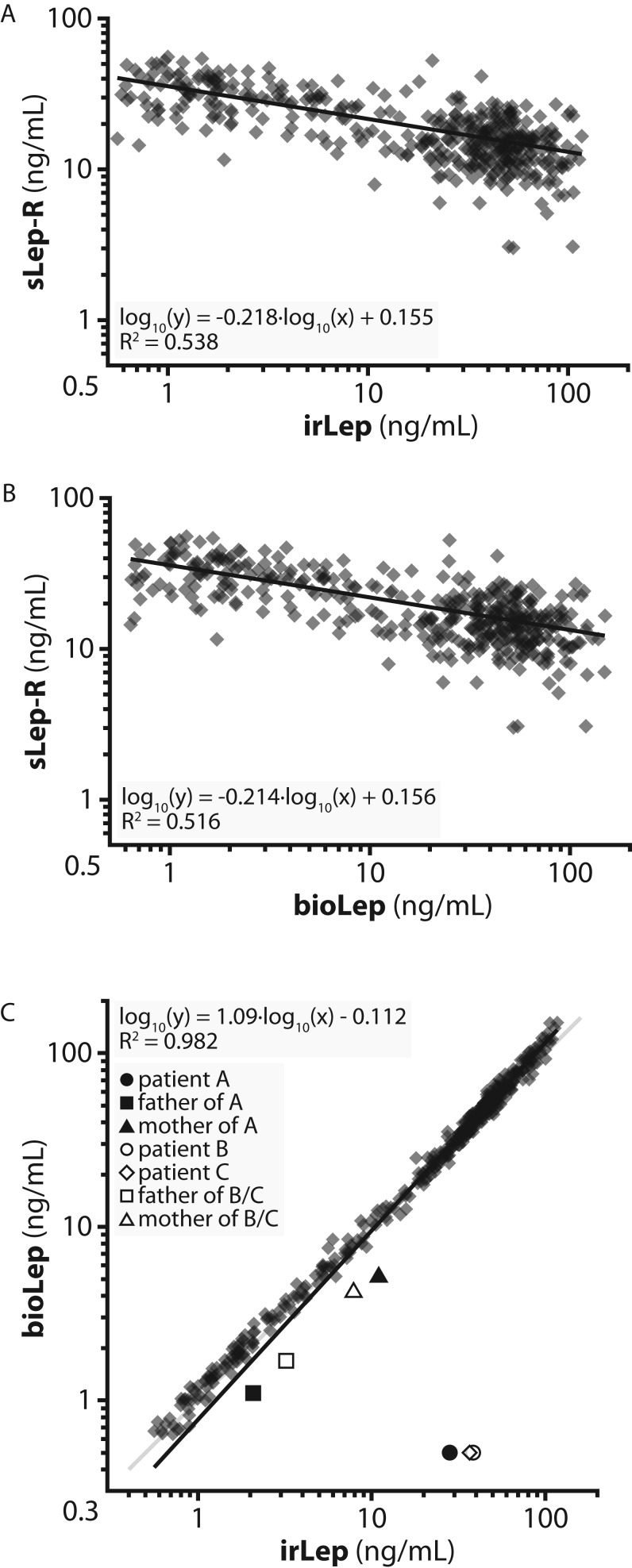
(A and B) Relationship between soluble leptin receptor (sLep-R) and irLep concentrations as well as sLep-R and bioLep concentrations in sera of a clinical cohort of lean and obese children and adults (*n* = 409, see ‘Subjects and methods’ section). (C) Relationship between serum bioLep and irLep concentrations in a clinical cohort of lean and obese children and adults (*n* = 409, see ‘Subjects and methods’ section) and in the three patients with confirmed bioinactive leptin. The patients were not included in regression analysis.

In the sera of the clinical cohort (*n* = 409), sLep-R concentrations in the range of 2.5–55.8 ng/mL were measured. There was a weak inverse correlation between concentrations of sLep-R and irLep as well as between concentrations of sLep-R and bioLep (Fig. 1A and B).

### Clinical validation of bioLep

Serum concentrations of leptin as determined by means of both irLep and bioLep assays from a cohort of lean and obese children and adults (*n* = 409, see ‘Subjects and methods’ section) were compared.

As illustrated in Fig. 1C, bioLep values were highly correlated to irLep values: (bioLep: median: 33.1 ng/mL; range: 0.6–150.0 ng/mL; irLep: median of 31.9 ng/mL; range: 0.6–117.2 ng/mL). The ratio of bioLep to irLep levels was 1.07 ± 0.11 (range 0.80–1.41).

In contrast, bioLep concentrations in sera of the patients with bioinactive leptin were below the detection limit, whereas the corresponding irLep concentrations were high (Table 3). The irLep concentrations were in or above the reference range of the assay (15) and seemed to be appropriate for the fat mass of these patients.

**Table 3:** Clinical characteristics and leptin levels of patients with bioinactive leptin and their parents.

	Leptin mutation	Age (years)	BMI (kg/m2)	bioLep (ng/mL)§	irLep (ng/mL)$	bioLep/irLep
Patients						
Patient A (male)	D100Y homozygous	2	44.6	<0.5	28.2	n.a.
Patient B (male)	N103K homozygous	6	35.2	<0.5	38.4	n.a.
Patient C (female)	N103K homozygous	9	39.6	<0.5	36.7	n.a.
Parents						
Father of A	D100Y heterozygous	35	25.9	1.1	2.1	0.53
Mother of A	D100Y heterozygous	31	26.1	5.3	11	0.48
Father of B/C	N103K heterozygous	41	23.8	1.7	3.2	0.53
Mother of B/C	N103K heterozygous	41	26.0	4.3	7.9	0.55

n.a.: not available as the bioLep was below the linear assay range; ^§^2.5–97.5 centile reference range: 11–92 ng/mL of severely obese children; ^$^2.5–97.5 centile reference range: 10–81 ng/mL of severely obese children (*n* = 26, age (mean ± s.d.: 11.0 ± 4.8 years), BMI (mean ± s.d.: 35.7 ± 7.7 kg/m²)).

When we compared the serum concentrations of irLep in the patients with bioinactive leptin to those of a subgroup of children who were part of the clinical cohort described but with extreme obesity as defined by a BMI >99.5 percentile (16) (*n* = 26, age (mean ± s.d.: 11.0 ± 4.8 years), BMI (mean ± s.d.: 35.7 ± 7.7 kg/m^2^)), we also observed that irLep concentrations of the patients lay within the expected range for their gender and BMI (Table 3).

In addition, the concentrations of bioLep in sera from heterozygous carriers of leptin gene mutations (parents of our patients with genetically proven bioinactive leptin) were determined. The bioLep concentrations in these sera were approximately half of the irLep concentrations resulting in bioLep-to-irLep ratios ranging from 0.48 to 0.55 (Table 3).

The changes in the serum concentrations of irLep and bioLep in patients with biologically inactive leptin were longitudinally assessed before and during replacement therapy with recombinant human methionyl-leptin (metreleptin). As shown in Table 4, hormone replacement resulted in increasing concentrations of bioLep during the course of treatment. Notably, after the start of hormone replacement, a continuous decrease in concentrations of irLep was observed in patients with a biologically inactive hormone. An increase in concentrations of bioLep was also observed in patients with classical leptin deficiency during treatment with metreleptin (Table 4).

**Table 4:** Serum levels of bioactive (bioLep) and immunoreactive (irLep) leptin in patients with biologically inactive leptin (patients A, B, C) and classical leptin deficiency (patients D and E) before and during replacement with metreleptin.

			Time on metreleptin*			
Patients	Parameter	Dimension	Start	T1	T2	T3
Patient A	bioLep	(ng/mL)	<0.5	<0.5	0.6	1.2
	irLep	(ng/mL)	28.2	13.4	7.0	2.6
	Fat mass	(kg)	22.0	20.7	14.5	6.9
	Fat mass	(% of total)	53.4	54.2	47.1	26.2
	Metreleptin dose	(mg/kg LBM/day)	0.015	0.032	0.035	0.028
Patient B	bioLep	(ng/mL)	<0.5	<0.5	0.8	1.1
	irLep	(ng/mL)	38.4	28.8	21.2	8.5
	Fat mass	(kg)	25.6	n.d.	n.d.	16.1
	Fat mass	(% of total)	51.8	n.d.	n.d.	42.1
	Metreleptin dose	(mg/kg LBM/day)	0.028	0.028	0.028	0.030
Patient C	bioLep	(ng/mL)	<0.5	<0.5	0.6	0.9
	irLep	(ng/mL)	36.7	35.5	29.3	16.9
	Fat mass	(kg)	39.5	n.d.	n.d.	35.0
	Fat mass	(% of total)	52.3	n.d.	n.d.	52.1
	Metreleptin dose	(mg/kg LBM/day)	0.029	0.029	0.029	0.165
Patient D	bioLep	(ng/mL)	<0.5	<0.5	3.1	4.5
	irLep	(ng/mL)	0.1	0.5	2.2	3.9
	Fat mass	(kg)	12.3	n.d.	6.3	5.9
	Fat mass	(% of total)	49.3	n.d.	31.3	28.9
	Metreleptin dose	(mg/kg LBM/day)	0.031	0.031	0.042	0.053
Patient E	bioLep	(ng/mL)	<0.5	0.5	8.2	11.3
	irLep	(ng/mL)	<0.5	<0.5	4.1	6.8
	Fat mass	(kg)	49.3	39.8	31.1	28.0
	Fat mass	(% of total)	50.1	44.9	39.3	36.0
	Metreleptin dose	(mg/kg LBM/day)	0.023	0.023	0.023	0.023

* See ‘Subjects and methods’ section for exact months on metreleptin.

LBM, lean body mass.

## Discussion

Classical and functional leptin deficiency are rare diseases, and only a small number of patients has been documented in the literature so far (6, 10, 11). Early-onset, extreme obesity due to biologically inactive leptin is a new disease entity characterized by high immunoreactive levels of circulating leptin, but a reduced bioactivity of the hormone caused e.g. by defects in receptor binding. However, most patients with bioinactive leptin are presently likely to remain undiagnosed as so far, only the immunoassay detecting total immunoreactive leptin is used as part of the recommended work-up of severe early-onset obesity. Hence, the diagnosis in first patients described with bio-inactive leptin as the cause of extreme obesity had to be established by complex molecular genetic investigations (10).

As extreme obesity due to bioinactive leptin due to poor receptor binding – like in leptin deficiency – is a treatable condition, the diagnosis should be confirmed or excluded in any suspected case as early in life as possible. Here, we established and validated a method to measure only the bioactive fraction (bioLep) of total serum leptin capable to bind to the receptor. We validated the bioLep assay clinically in a large cohort with a wide age and BMI range and demonstrated that it is capable of measuring leptin concentrations in serum as reliably and accurately as conventional immunoassays. Moreover, we verified that the bioLep assay is able to detect clinical cases of bioinactive leptin. In addition, we were able to show that individuals heterozygous for bioinactive leptin mutations have ratios of bioactive leptin to immunoreactive leptin (bioLep/irLep) clearly distinguishable from homozygous wild-type individuals. The bioLep assay presented here facilitates the diagnosis of both classical and functional leptin deficiency due to poor receptor binding enormously.

The follow-up data on leptin levels in the three patients with biologically inactive leptin after the start of metreleptin treatment revealed a continuous decrease in irLep levels. Mechanistically, the therapy-associated weight loss and decreased adipose tissue mass might be central to this phenomenon. Interestingly, leptin has been proposed to regulate its own expression in adipocytes (17). We thus postulate a direct metreleptin-induced downregulation of the endogenous expression of mutated leptin by the patients’ adipocytes as a mechanism contributing to the observed fall in circulating irLep.

We showed here that the bioLep assay is capable of detecting individuals with heterozygous leptin gene mutations by calculating the ratio of bioactive leptin to immunoreactive leptin, the latter being measured by a conventional assay that has to be calibrated equally. Interestingly, the heterozygous parents of our patients displayed bioLep/irLep ratios of approximately 0.5, suggesting that overall both alleles are expressed at even levels.

Phenotypic characteristics of individuals with heterozygous leptin mutations leading to classical leptin deficiency have been described and discussed earlier (18). The phenotype of individuals heterozygous for mutations resulting in bioinactive leptin, however, has not been studied in detail. The four individuals with this condition presented here were not extremely obese and had no reported hyperphagia.

By determining the bioLep/irLep ratio in large cohorts, it will now be possible to identify additional individuals heterozygous for biologically inactive leptin due to impaired receptor binding. Screening studies in representative cohorts will help to estimate the frequency of heterozygosity of leptin gene mutations in distinct populations. Subsequent phenotyping studies of heterozygous individuals will help to better delineate the phenotype of individuals heterozygous for bioinactive leptin mutations.

Nevertheless, there may be factors influencing the results of this study. Our study has certain analytical restrictions. Firstly, measured values for bioLep and the hormone receptor interaction in our assay may be influenced by the competition of different amounts of endogenous leptin-binding proteins. However, the association of the major leptin-binding protein sLep-R with bioLep and irLep is only marginal. In addition, the saturation of increasing concentrations of sLep-R has negligible effects on the target levels of bioLep as well as irLep in the respective assays. Furthermore, within the physiological range of sLep-R, we observed no clinically relevant interference.

Secondly, it may be possible that the binding procedure of leptin molecules to the extracellular domain of the leptin receptor in the bioLep assay does not mirror the physiological receptor binding process perfectly. The sLep-R molecule used for the bioLep assay originates from HEK293 cells and represents a chimeric protein that may have binding characteristics differing from those of the endogenous leptin receptor *in vivo*. However, a relevant effect influencing the results could be excluded as the results of measured serum bioLep and irLep concentrations in our clinical cohort were largely comparable.

Thirdly, when comparing data of two analytical methods, the obtained results are affected by the imprecision or variance of these methods. Accordingly, when measuring both bioLep and irLep in samples of the control cohort a median ratio of 1.07 ranging from 0.80 to 1.41 was noted. The causes of the minor difference between the measurement results when comparing both assays still remain unclear.

Finally, it needs to be considered that the bioLep assay developed here may not be able to detect all existing bioinactive leptin mutants. Theoretically, leptin mutants still capable of binding but not activating the leptin receptor might exist.

In summary, we have shown that the new immunofunctional bioLep assay presented in this study is able to detect patients with reduced leptin bioactivity due to poor receptor binding resulting from either homozygous or heterozygous mutations in the leptin gene that eliminate leptin receptor binding. This functional leptin deficiency constitutes a treatable condition – consequently, diagnosis should take place as early in life as possible. The bioLep assay is a time- and cost-effective diagnostic tool that will accelerate the work-up of suspicious patients and ultimately allow proper therapy to be initiated where applicable.

## Declaration of interest

M W received honoraria from Mediagnost for lectures. B F, L P are employees at Mediagnost. M R received honoraria from Mediagnost for lectures. M S, J K, J v S, A M, S B, K K, P F P, J B F, B M, P G have nothing to disclose.

## Funding

This work was supported by Grant BMBF 01GI1120A from the German Federal Ministry of Education and Research. J B F was supported by the International Graduate School in Molecular Medicine Ulm.

## Author contribution statement

M W, L P, J K, M R, B F conceived and designed the study. M W, J v S, K K performed the clinical measurements. J K, L P, M S, B F performed the laboratory measurements. M W, A M, S B, J B F, L P, J K analyzed the data. M W, A M, J K wrote the first draft of the manuscript. All authors contributed to the writing of the manuscript and agree with the final manuscript and conclusions.
